# Questions concerning the proximal origin of SARS‐CoV‐2

**DOI:** 10.1002/jmv.26478

**Published:** 2020-12-30

**Authors:** Murat Seyran, Damiano Pizzol, Parise Adadi, Tarek M. A. El‐Aziz, Sk. Sarif Hassan, Antonio Soares, Ramesh Kandimalla, Kenneth Lundstrom, Murtaza Tambuwala, Alaa A. A. Aljabali, Amos Lal, Gajendra K. Azad, Pabitra P. Choudhury, Vladimir N. Uversky, Samendra P. Sherchan, Bruce D. Uhal, Nima Rezaei, Adam M. Brufsky

**Affiliations:** ^1^ Infection, Malignancy and Autoimmunity (NIIMA), Universal Scientific Education and Research Network (USERN), Doctoral Studies in Natural and Technical Sciences (SPL 44)xs University of Vienna Vienna Austria; ^2^ Infection, Malignancy and Autoimmunity (NIIMA) Universal Scientific Education and Research Network (USERN) Vienna Austria; ^3^ Italian Agency for Development Cooperation ‐ Khartoum Al Amarat Sudan; ^4^ Department of Food Science University of Otago Dunedin New Zealand; ^5^ Department of Cellular and Integrative Physiology University of Texas Health Science Center at San Antonio San Antonio Texas USA; ^6^ Department of Zoology, Faculty of Science Minia University El‐Minia Egypt; ^7^ Department of Mathematics Pingla Thana Mahavidyalaya Maligram West Bengal India; ^8^ CSIR‐Indian Institute of Chemical Technology Hyderabad Telangana India; ^9^ Kakatiya Medical College/MGM‐Hospital, DME/TSPSC, Hyderabad Warangal Telangana India; ^10^ PanTherapeutics Lutry Switzerland; ^11^ School of Pharmacy and Pharmaceutical Science Ulster University Coleraine Northern Ireland UK; ^12^ Department of Pharmaceutical Sciences, Faculty of Pharmacy Yarmouk University Irbid Jordan; ^13^ Division of Pulmonary and Critical Care Medicine Mayo Clinic Rochester Minnesota USA; ^14^ Department of Zoology Patna University Patna India; ^15^ Applied Statistics Unit Indian Statistical Institute Kolkata West Bengal India; ^16^ Department of Molecular Medicine, Morsani College of Medicine University of South Florida Tampa Florida USA; ^17^ Department of Environmental Health Sciences Tulane University New Orleans Louisiana USA; ^18^ Department of Physiology Michigan State University East Lansing Michigan USA; ^19^ Center for Immunodeficiencies, Pediatrics Center of Excellence, Children's Medical Center Tehran University of Medical Sciences Tehran Iran; ^20^ Network of Immunity in Infection, Malignancy and Autoimmunity (NIIMA) Universal Scientific Education and Research Network (USERN) Tehran Iran; ^21^ Department of Medicine, Division of Hematology/Oncology UPMC Hillman Cancer Center Pittsburgh Pennsylvania USA

**Keywords:** coronavirus, fusion protein, genetic variability, mutation

## 1 SUMMARY

There is a consensus that severe acute respiratory syndrome coronavirus 2 (SARS‐CoV‐2) originated naturally from bat coronaviruses (CoVs), in particular RaTG13. However, the SARS‐CoV‐2 host tropism/adaptation pattern has significant discrepancies compared with other CoVs, raising questions concerning the proximal origin of SARS‐CoV‐2. The flat and nonsunken surface of the sialic acid‐binding domain of SARS‐CoV‐2 spike protein (S protein) conflicts with the general adaptation and survival pattern observed for all other CoVs. Unlike RaTG13, SARS‐CoV‐2 recombination presumably occurred between the S1/S2 domains of S protein enabling host furin protease utilization. Although millions of recorded cases have been recorded globally, SARS‐CoV‐2 S protein does not have any apparent further recombination, placing it in conflict with the recombination models of other CoVs. Similarly, the S protein receptor‐binding domain (RBD) of SARS‐CoV‐2 has not accumulated high‐frequency nonsynonymous substitutions, differentiating SARS‐CoV‐2 from other CoVs that have positive selection/adaptation mutations in their RBDs.

## 2 DISCUSSION

Andersen et al.^1^ documented the possible natural origin of SARS‐CoV‐2 from BatCoV RaTG13.^2^ SARS‐CoV‐2 is the seventh zoonotic CoV virus capable of infecting humans, but the first and only human coronavirus (HCoV) with pandemic potential.^3^ Bat or rodent CoVs demonstrate certain specific changes in the S protein RBD, as well as the S protein glycan‐binding N‐terminal domain (NTD), during host tropism/adaptation.^4^, ^5^ SARS‐CoV‐2, unlike other CoVs, does not have those signature changes, suggesting that these RBD and NTD subdomains are of very recent origin.

The “Canyon Hypothesis” explains the development of canyons, depression zones, or cavities on the surfaces of influenza virus, human rhinovirus, and Meningo viruses.^6^ In CoVs (except SARS‐CoV‐2), the S protein NTD domain has several predicted glycan‐binding domains, with a common feature being the hidden localization of these glycan‐binding domains to cavities to limit their access to antibodies and immune cells.^5^ This pattern of CoVs is thought to be an evolutionary measure to restrict the recognition of these active sites by host immune system.^4^

HCoVs can evade detection by host glycan‐binding immune receptors. Comparative genomic analysis of six HCoVs with their corresponding native bat or rodent CoVs suggests compatibility with the “Canyon Hypothesis” resulting from various adaptive S protein NTD nonsynonymous mutations near or at the glycan‐binding domain which are predicted to result in these NTD domains being hidden below the protein surface.^5^ The predicted flat, nonsunken pattern of the SARS‐CoV‐2 S protein NTD glycan‐binding domains conflicts with this evolutionary host tropism/adaptation strategy.^7^

A template‐switching mechanism is presumably responsible for the high rate of RNA recombination in CoVs. In host cells, CoV RNAs show discontinuous RNA synthesis materialized by pauses of the RNA‐dependent complex and subsequent jumps to downstream template acceptor sequences. This process results in subgenomic minus‐strand RNAs which serve as templates for subgenomic messenger RNAs. Due to the mechanistic similarity to recombination, this process might be at the origin of recombinant CoVs co‐opting other CoV or even host‐related sequences.^8^ Instances include the mouse hepatitis coronavirus S protein NTD sialic acid‐binding domain, likely arising from recombination of viral RNA with human galectin RNA sequences.^8^

The furin recognition motif present at the SARS‐CoV2 S1/S2 junction has no analogy in other “linage B” beta‐coronaviruses, including neither pangolin‐CoV nor RaTG13.^1^ This indicates that the S protein S1/S2 junction is not a hot spot for RNA recombination termination that depends on a pattern swapping templates (copy‐choice).^8^ In addition, clinical isolates of SARS‐CoV‐2 S protein have not indicated any further recombination in this S1/S2 area, suggesting that the addition of a motif for S1/S2 site furin cleavage constituted a unique recombination occurrence. Finally, the CoV‐unique insertion of four aminoacids creating a novel RRAR furin cleavage site introduces two arginine codons CGG–CGG, whose usage is extremely rare in CoVs, further supporting the hypothesis of a unique recombination occurrence.

HCoVs have high‐frequency “hot spots” for nonsynonymous amino acid replacements that can possibly create a positive selection for host tropism/adaptation, resistance to neutralizing antibodies, or immune evasion.^2^ Interestingly, clinical SARS‐CoV‐2 isolates to date have only a single high‐frequency nonsynonymous mutation, D614G, in their S protein.^9^ Based on currently known mutation rates and patterns in clinical isolates of SARS‐CoV‐2, the S protein does not appear to be a mutational “hot spot” for SARS‐CoV‐2, unlike other human CoVs.

SARS‐CoV‐2 is the seventh HCoV, but the first HCoV with pandemic potential. SARS‐CoV disappeared without a pandemic, and MERS‐CoV is mostly endemic to the Arabian Peninsula with some additional limited traveler infections resulting in outbreaks in South Korea.^3^, ^4^ These unique features of SARS‐CoV‐2 raise several questions concerning the proximal origin of the virus that require further discussion.

## CONFLICT OF INTERESTS

The authors declare that there are no conflict of interests.

## AUTHOR CONTRIBUTIONS

Murat Seyran conceived the study. Adam M. Brufsky, Vladimir N. Uversky, Amos Lal, and Kenneth Lundstrom provided critical review. Nima Rezaei, Damiano Pizzol, Amos Lal, Samendra P. Sherchan, Vladimir N. Uversky, Kenneth Lundstrom, Tarek M. A. El‐Aziz edited the article, and Murat Seyran, Parise Adadi, and Tarek M. A. El‐Aziz formatted the article. All authors of the consortium interpreted the results and approved the final version for submission.

## KEYWORDS

coronavirus, fusion protein, genetic variability, mutation
